# Modeling of Microvascular Permeability Changes after Electroporation

**DOI:** 10.1371/journal.pone.0121370

**Published:** 2015-03-20

**Authors:** Selma Corovic, Bostjan Markelc, Mitja Dolinar, Maja Cemazar, Tomaz Jarm

**Affiliations:** 1 University of Ljubljana, Faculty of Electrical Engineering, Laboratory of Biocybernetics, Trzaska cesta 25, SI-1000 Ljubljana, Slovenia; 2 Institute of Oncology Ljubljana, Department of Experimental Oncology, Zaloska 2, SI-1000 Ljubljana, Slovenia; 3 University of Primorska, Faculty of Health Sciences, Polje 42, SI-6310 Izola, Slovenia; Medical University of Graz, AUSTRIA

## Abstract

Vascular endothelium selectively controls the transport of plasma contents across the blood vessel wall. The principal objective of our preliminary study was to quantify the electroporation-induced increase in permeability of blood vessel wall for macromolecules, which do not normally extravasate from blood into skin interstitium in homeostatic conditions. Our study combines mathematical modeling (by employing pharmacokinetic and finite element modeling approach) with *in vivo* measurements (by intravital fluorescence microscopy). Extravasation of fluorescently labeled dextran molecules of two different sizes (70 kDa and 2000 kDa) following the application of electroporation pulses was investigated in order to simulate extravasation of therapeutic macromolecules with molecular weights comparable to molecular weight of particles such as antibodies and plasmid DNA. The increase in blood vessel permeability due to electroporation and corresponding transvascular transport was quantified by calculating the apparent diffusion coefficients for skin microvessel wall (D [μm^2^/s]) for both molecular sizes. The calculated apparent diffusion coefficients were D = 0.0086 μm^2^/s and D = 0.0045 μm^2^/s for 70 kDa and 2000 kDa dextran molecules, respectively. The results of our preliminary study have important implications in development of realistic mathematical models for prediction of extravasation and delivery of large therapeutic molecules to target tissues by means of electroporation.

## Introduction

Electroporation (EP) is a physical method used for controlled increase of cell membrane permeability for large extracellular molecules which otherwise cannot enter the cytosol in homeostatic conditions [1,2]. In vivo electroporation is successfully used in a wide range of medical applications such as electrochemotherapy, medical applications based on non-thermal irreversible electroporation, electroporation-based gene therapy (i.e. electrogene therapy), DNA vaccination, and dermal and transdermal delivery of therapeutic agents [3–6]. Numerous clinical and preclinical studies demonstrated efficacy of electrochemotherapy for treatment of tumors of different histologies with both local and systemic administration of anticancer drugs [7–10]. Recent clinical trials of gene therapy using electroporation for gene electrotransfer demonstrated encouraging results also for therapeutic DNA delivery to different types of tissues, including skin, muscle, liver and tumor [11–13]. Similarly, clinical trials of electroporation-based DNA vaccination have shown promising results for DNA delivery in different tissues [14,15]. For effective DNA vaccine delivery skin and muscle have been proposed as tissues of choice [16].

It is well known that the outcome of electroporation-based therapies also strongly depends on effects of electroporation on tissue vasculature [17]. First, electroporation has blood flow modifying effects in both normal and neoplastic tissues [18–23]. Second, electroporation induces structural changes in cell-to-cell junctions of endothelial cells and structural alterations of endothelial lining of electroporated blood vessels [24,25]. In addition, recent *in vivo* studies demonstrated that electroporation increases the permeability of blood vessels’ wall, therefore facilitating the extravasation of macromolecules from blood vessels into the surrounding tissue [21–23]. The level of blood vessel wall permeabilization might therefore be of particular importance for effective systemic delivery of macromolecular therapeutic agents to the target tissue.

Currently, only realistic mathematical modeling approaches have been used to predict *in vivo* electroporation-facilitated permeabilization of target cells (e.g tumor cells in case of ECT) based on assumed electric field distribution in bulk tissue [26–28]. However, several studies have highlighted the need for direct measurement and development of corresponding mathematical models of electroporation-facilitated molecular transport (e.g. transport of therapeutic agents to the target cells) for improved predictions of *in vivo* cell and tissue electroporation efficacy and subsequent outcome of electroporation based therapies [29–31]. To the best of our knowledge no previous *in vivo* investigation of electroporation-enhanced transvascular transport of therapeutic agents has been reported.

In general, two modes of transport are involved in macromolecular transport across microvascular wall and through the extravascular space; convection and diffusion. The relative contributions of convective and diffusive components of transvascular transport are still a subject of extensive research and different models have been proposed and used to quantify them [32–36]. The most common approaches to study and quantify transvascular transport usually employ intravital confocal microscopy to measure directly intra and extravascular concentrations of fluorescently labeled molecules and use Kedem-Katchalsky equation to estimate microvascular apparent permeability [37,38]. In this preliminary work we used intravital stereo microscopy which did not allow us to measure the 3D spatial distribution of concentration. Due to this limitation we approached the quantification of dextran extravasation by modeling the fundamental process of molecular diffusion by solving the partial differential equation (Fick’s law) for transvascular as well as interstitial transport by means of finite element method (FEM).

The principal aim of our study was to investigate and quantify the electroporation facilitated extravasation of macromolecules (i.e. fluorescently labeled dextrans (FD) with molecular weights of 70 kDa and 2000 kDa) which do not extravasate readily from normal blood vessels into surrounding tissue in homeostatic condition [39]. We combined mathematical modeling (pharmacokinetic and FEM models) with *in vivo* measurements (intravital fluorescence microscopy imaging of a mouse skin in a dorsal window chamber (DWC)) to quantify the electroporation facilitated increase in microvascular permeability for both sizes of dextran. The acquired time-series of images allowed us to determine the spatial and temporal profile of fluorescence intensity of FD inside and outside skin microvessels and thus to quantify the extent of FD extravasation from microvessels. Fitting the parameters of our mathematical model to experimental data showed higher microvascular permeability after electroporation for smaller FD molecules compared to the larger ones. The results of this preliminary study contribute to clearer understanding of mechanisms of extravasation and transport of macromolecules throughout the tissue involved in efficacy of electroporation-based treatments which require large therapeutic molecules to be transported across the blood vessel wall to the target cells.

## Materials and Methods

### In vivo experiments

#### Reagents

The 70 or 2000 kDa fluorescein isothiocyanate-labeled dextrans (Sigma-Aldrich) with the polydispersity of ~1.5 [40] were resuspended in phosphate-buffered saline (PBS). The fluorescent dextrans (FD) were then washed two times for 3 h through 30-kDa or 1000 kDa Vivaspin ultrafiltration spin columns (Sartorius Stedim Biotech GmbH) to remove any free fluorochromes or low molecular weight contaminants. The high molecular weight component was then resuspended in phosphate-buffered saline (PBS) at final concentration of 37.5 mg/ml and a bolus injection of 100 μl of this suspension was administered intravenously (intraorbitally) to mice in the experiments.

#### Mice

Adult Balb/c mice (Harlan, Italy) were held in a specific pathogen-free animal colony at controlled temperature and humidity with 12-h light/dark cycles. Food and water were provided *ad libitum*. Before the experiments, mice were subjected to an adaptation period of 14 days. Experiments were performed on female mice, 12–14 weeks old and weighing 20–25 g. All animal experiments were conducted in accordance with the guidelines for animal experiments of the EU Directives and the permission obtained from the Ministry of Agriculture and the Environment of the Republic of Slovenia (Permission No. 34401–12/2009/6 which was specifically given for this study based on the approval of the National ethics committee for experiments on laboratory animals). The National ethics committee is under the auspices of Ministry of Agriculture and Environment of the Republic of Slovenia.

On each mouse (out of 6 mice used) two consecutive experiments were performed. For the control measurement only FD was injected and no electric pulses were delivered. Two days later, when the initial FD was washed out of the system, FD was injected again, followed after 12 minutes by the treatment with electric pulses (i.e. electroporation).

#### Preparation of the dorsal window chamber (DWC) in mice

The DWC was surgically implanted on the back of mice as described previously [41]. Briefly, the DWC implantation was carried out under general anesthesia using an intraperitoneal injection of ketamine (1 mg/ml, Narketan, Vetoquinol AG), xylazine (5 mg/ml, Chanazine, Chanelle Pharmaceuticals Manufacturing Ltd.) and acepromazyne (0.4 mg/ml, Promace, Fort Dodge Animal Health). The back of the mouse was first shaved and then depilated (Veet, Reckitt Benckiser Group). During the surgery mice were kept on a heating pad to prevent hypothermia and an aseptic technique was used throughout the surgical procedure. The DWC (APJ Trading Co.) consisting of two titanium frames sandwiched an extended double layer of dorsal skin with the use of stainless steel screws and sutures. Then, one layer of the skin (12-mm diameter) was surgically removed. The exposed inner surface of the remaining skin layer was covered with a glass coverslip and secured with a stainless steel clip, thus creating an observation window enabling visual access to the skin vasculature. After the surgery and for the following 2 days analgesia was provided by intramuscular injection of butorphanol (0.3 mg/kg, Torbugesic, Fort Dodge Animal Health) to alleviate possible postsurgical pain.

#### Electroporation

Electroporation (EP) was performed 4–10 days after the implantation of DWC. Parameters of electric pulses commonly used in clinical electrochemotherapy and clinical electrogene therapy were applied (i.e. 8 square-wave electric pulses with the voltage-to-distance ratio of 1300 V/cm (amplitude 780 V), duration of 100 μs and frequency of 1 Hz) [11,42]. Pulses were generated by Cliniporator (IGEA S.r.l., Italy) and delivered by two parallel stainless steel plate electrodes (30 mm long, 6 mm wide) placed 6 mm apart. The electrodes were placed on the skin on the opposite side of the DWC glass coverslip, where the epidermis was intact. ECG conductive gel (P. J. Dahlhausen & Co. GmbH) was used to obtain good electrical contact between the electrodes and the skin. The electrical parameters of electric pulses were monitored on the generator to guarantee that all pulses were successfully delivered.

#### Intravital microscopy setup and image acquisition

**Fig 1:**
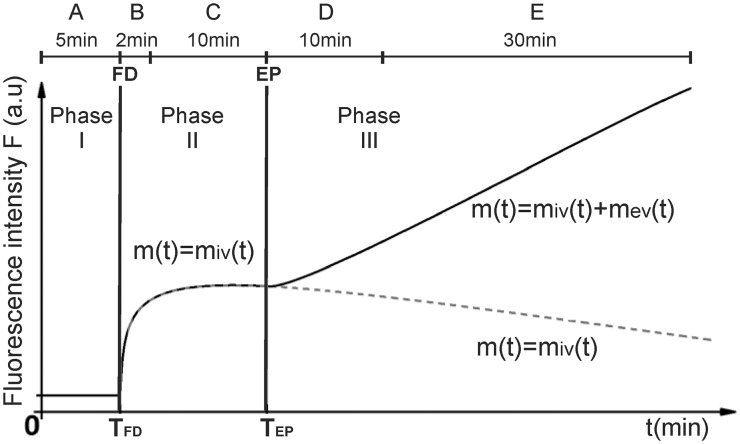
Timeline of image acquisition and temporal profile of fluorescence intensity *F*(*t*) obtained within the experiments. A, B, C, D, E—intervals with different rates of image acquisition, FD—intraorbital injection of fluorescent dextran FD, EP—electroporation (i.e. delivery of electric pulses). Temporal profile of *F*(*t*) within the three phases, obtained within the experiments: Phase I (interval A)- Acquisition of *F*(*t*) observed in tissue in absence of dextran; Phase II (intervals B and C)—Acquisition of *F*(*t*) and the corresponding dextran in blood vessels *m*_*iv*_ and Phase III (intervals D and E)—Acquisition of *F*(*t*) and corresponding dextran content in blood vessels (*m*_*iv*_) and the corresponding the dextran extravasated into the tissue (*m*_*ev*_). *T*_*FD*_ and *T*_*EP*_ stand for the time t at which the dextran FD was injected and electroporation EP was performed, respectively.

Intravital microscopy was carried out using a Carl Zeiss SteREO Lumar. V12 (Carl Zeiss, Germany) fluorescence stereomicroscope equipped with a NeoLumar S 0.8x objective (Carl Zeiss) and an MRc 5 digital camera (Carl Zeiss). Animals were anesthetized by inhalation anesthesia (Isofluran, Nicholas Piramal India Ltd.) and placed on a custom-designed holder which provided means for secure fixation of the DWC in a horizontal position. A time-series of images was acquired at different rates as follows (top of Fig. 1): A—one frame per 20 seconds for 5 minutes (FD suspension was injected intraorbitally at the end of this interval, *T*_*FD*_); B—one frame per 5 seconds for 2 minutes, starting 10 seconds after intraorbital injection of FD; C—one frame per 20 seconds for 10 minutes (EP pulses, were delivered at the end of this interval, at *T*_*EP*_); D—one frame per 20 seconds for 10 minutes; E—one frame per 2 min for 30 min. The images were stored and analyzed off-line with image analysis software AxioVision (Carl Zeiss) and Matlab software (MathWorks, Natick, ZDA). The same schedule was used in control experiments except that no EP pulses were delivered. Images were acquired at 40x magnification with the size of 1292x968 pixels, the resolution of 3.4 μm/pixel and with 16-bit depth.

#### Image analysis considerations

In our setup the FD fluorescence from the entire thickness of the skin layer was acquired. The images contained blood vessels of different sizes including microvessels (capillaries) and larger vessels of the skin. Presence of large vessels in the images would impose complications on mathematical modeling of FD extravasation since the arrangement and geometry of large vessels is not uniform and is specific to each treated mouse. The effect of electroporation on permeability of larger vessels’ wall is also size-dependent, which would require a different model (or at least different parameter values) for each vessel size and type. In addition, larger vessels respond to EP pulses with rapid and profound vasoconstriction resulting in a reduction in diameter of the vessels, and in a non-uniform and time-dependent recovery [21,23].

Therefore in order to focus on electroporation-induced changes in microvessels' wall permeability, we eliminated the areas containing larger vessels and their immediate surroundings from the acquired images and analyzed only the fluorescence intensity within the remaining areas containing the intra- and extravascular compartments of microcirculation (i.e. predominantly capillaries). The geometry and arrangement of normal skin capillaries can be considered uniform and the capillaries do not respond to EP pulses with vasoconstriction, therefore we were able to develop a generalized model of transvascular transport of FD which was applicable to all our experiments.

The image analysis and image masking procedure for removal of areas with larger vessels were described in detail previously [21,23]. Briefly, on the last image taken before the electroporation (the end of interval C in Fig. 1) a low-pass filter was applied to average out rapid changes in intensity and the resulting image was subtracted from the unfiltered original one. On this subtracted image a suitable threshold which most accurately discriminated between the large blood vessels and the tissue outside the large blood vessels was applied in order to obtain a binary mask of the large blood vessel network. This mask was then superpositioned onto the original image and the discrepancies between it and the blood vessels network on the original image were manually corrected. After superimposing the corrected mask on all the aligned images of the acquired time series in Matlab software, the mean average fluorescence intensity of pixels outside the masked region (corresponding to the extravascular compartment and the microcirculatory network) before and after electroporation was determined in order to obtain a temporal profile of the average fluorescence intensity *F*(*t*) for the whole region of interest.

#### Determination of *in vivo* dynamics of dextran extravasation

*In vivo* dynamics of FD amount inside and outside of microvessels *m*(*t*) was estimated for each interval from Fig. 1 separately based on the fluorescence profile *F(t)*. The assumptions and the relationship between *F*(*t*), *m*(*t*) and the average FD concentration *c*(*t*) in the region of interest are described in detail in S1 Text.

A schematic temporal profile of fluorescence intensity obtained in the experiments is shown in Fig. 1. Our image acquisition protocol provided separate sets of data for the three distinct phases with respect to the injection of FD and the application of EP pulses (Fig. 1). Phase I provided information about the background fluorescence intensity in the tissue before injection of FD. The averaged background fluorescence was subtracted from all images before any further processing. Phase II provided the data on FD content only inside the microcirculatory network because in homeostatic conditions in skin dextrans of molecular size 70 kDa and larger do not extravasate in significant amounts within 60 minutes after intravenous injection [39,43,44]. In control experiments (with no EP pulses) Phase III provided the continuation of the data from Phase II with FD remaining confined to intravascular compartment (dashed line) according to Equation (1). The negative slope of the dashed line at the and of Phase II and throughout Phase III was a result of clearance of FD from the blood (e.g. in kidneys). In case of EP pulse delivery, however, Phase III provided the data on the cumulative FD amount in both intra- and extravascular compartments due to electroporation-induced increased permeability of blood vessel walls and subsequent extravasation of FD (solid line), according to Equation (2).

$$m(t)=m_{iv}(t),\;T_{FD}<t<40\;\min$$ (1)

$$m(t)=\left\{ \begin{array}{l} m_{iv}(t),\;T_{FD}<t<T_{EP}\\ m_{iv}(t)+m_{ev}(t),\;T_{EP}<t<40\;\min \end{array}\right.$$ (2)

In Equation (1) and (2) *m*_*iv*_(*t*) and *m*_*ev*_(*t*) are the FD amounts inside microcirculatory vessels and in the extravascular space, respectively. *T*_*FD*_ is the start of the FD injection and the *T*_*EP*_ is the start of electroporation.

#### Mathematical model for analysis of experimental results

The experimentally obtained temporal profiles of 70 and 2000 kDa FD fluorescence intensity (Phase II and Phase III in Fig. 1) represented the input data for mathematical modeling. The modeling of transvascular transport was realized by using two interconnected models: a two-compartment pharmacokinetic model (described in detail in S2 Text) and a finite element method (FEM) model of transvascular diffusive transport described by Fick's law (Equation (S10), see S3 Text). However, to reflect the fact that the unknown influence of convection contributed to the estimated value of diffusion coefficient we are using the term "apparent" diffusion coefficient instead of diffusion coefficient throughout the paper.

The two-compartment pharmacokinetic model (see S2 Text) was used to estimate the temporal profile of FD concentration inside the capillaries *c*_*iv*_(*t*), which was then incorporated inside the FEM model built using Comsol Multiphysics software (Comsol AB, Stockholm, Sweden). The details and the underlying theoretical considerations for the FEM model are given in S3 Text, but the outline of the DWC skin model geometry is presented here.

**Fig 2:**
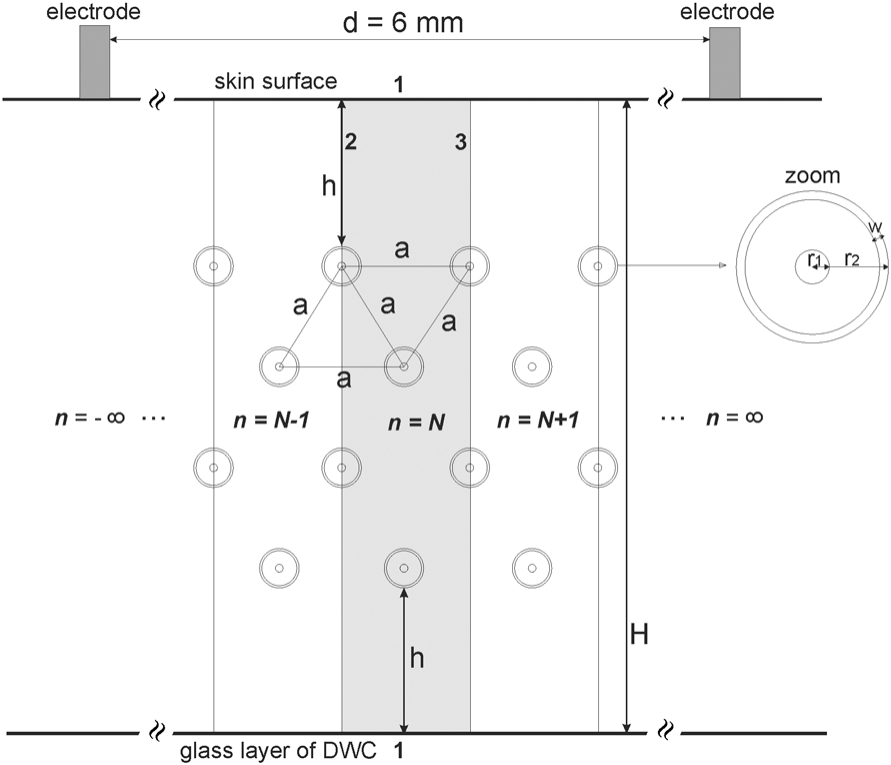
The modeled infinite geometry of skin and capillaries. Infinite geometry is created by infinitely repeating the grayed-out region which we realized in Comsol Multiphysics by applying boundary conditions of symmetry at the inner boundaries 2 and 3. The height of the skin layer was *H* = 300 μm. The radius of microvessels was *r* = 3.5 μm. The distance from the skin surface to the capillaries was *h* = 72 μm. The distance between the centers of neighboring capillaries was *a* = 60 μm. The thickness of capillary wall was *w* = 1 μm. The distance between electrodes was *d* = 6mm.

The geometry of FEM model was based on the data obtained from our histological measurements of arrangement and geometry of capillaries in the DWC skin preparations [21] (and unpublished data) and is shown in Fig. 2. The arrangement of the capillaries was assumed to be uniform within the region of interest (homogeneous distribution of capillaries is a well known characteristic of normal tissue [37]). Capillaries were modeled as infinitely long and parallel tubes, which allowed us to use 2D instead of 3D geometry. Further simplification of the model was possible due to the fact that the region of interest in the images was selected far enough from the position of the electrodes that the entire region of interest could be considered uniformly electroporated. This allowed us to model the entire region of interest by modeling only a small part of it and by applying the appropriate boundary conditions to it. These simplifications were crucial for decreasing the computational time of our optimization simulations.

The height of the skin layer (i.e. from the skin surface to the DWC glass cover) was H = 300 μm. The microvessels with the radius *r* = 3.5 μm were arranged in four parallel layers in the center of the skin at the distance *h* = 72 μm from both surfaces so that the centers of the capillaries were positioned in the corners of equilateral triangles as shown in Fig. 2. The distance between the neighboring capillaries (i.e. centers of cylinders) was *a* = 60 μm which corresponds to the average distance measured between adjacent microvessels in the examined mouse skin. The capillary wall was modeled as a layer with the thickness of *w* = 1 μm surrounding each cylinder.

#### Determination of apparent diffusion coefficient

**Fig 3:**
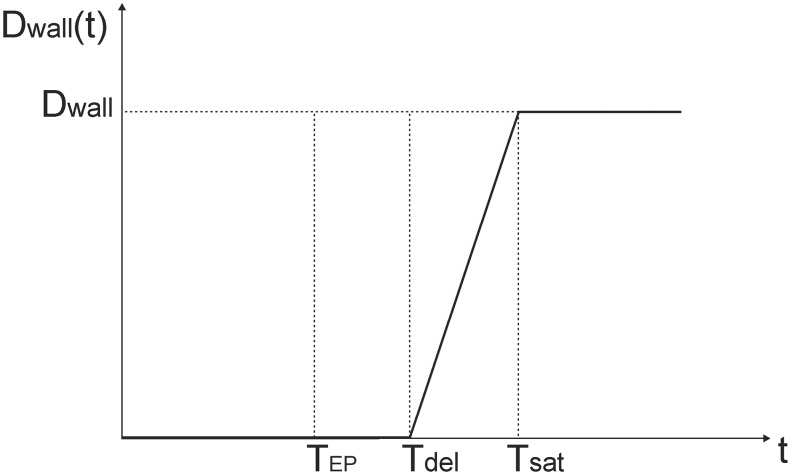
The time-dependent function of apparent diffusion coefficient *D*_*wall*_(t) of electroporated microvessel wall. *T*_*EP*_ is the start of electroporation. The delay time *T*_*del*_ and the saturation time *T*_*sat*_ together define the slope of the linearly increasing part of *D*_*wall*_(t) and the final (saturation) value of *D*_*wall*_(t): *D*_*wall*_ for t ≥ *T*_*sat*_.

From our own experimental observations of FD extravasation within this study and based on the dynamics of induced structural changes in cell-to-cell junctions of endothelial cells and structural alterations of endothelial lining after application of EP pulses [24] it followed that the microvascular permeability increase was not an instantaneous phenomenon. Therefore the apparent diffusion coefficient was modeled as a time dependent function *D*_*wall*_(*t*) shown in Fig. 3, where *T*_*EP*_ corresponded to the moment of application of EP pulses (see also Fig. 1), In our model the initial value of the apparent diffusion coefficient *D*_*wall*_(*t*) was considered to be zero prior to *T*_*EP*_ and started to increase with a delay at *T*_*del*_ after application of EP. *D*_*wall*_(*t*) reached its final "saturated" value at *T*_*sat*_. It was asumed that the final value of *D*_*wall*_(*t*) remained constant until the end of the observation period. Values of parameters *T*_*del*_, *T*_*sat*_ and the final value of *D*_*wall*_ were all determined during the optmization procedure in which the FEM model of diffusion was fitted to the experimental data. The optimization procedure was based on the so-called genetic algorithm previously developed by our group for a different purpose and described in detail elsewhere [26]. The genetic algorithm-based optimization was used in order to avoid the convergence problems commonly occurring when using other optimization approaches [45]. Due to the small size of the samples we report our results as individual data and refer to the median values when summarising the results.

The diffusion coefficient of the extravascular space *D*_*tiss*_ in our models was set to a constant value, which we estimated based on the data from the literature [34,46]. Since the estimation of *D*_*tiss*_ value was unreliable, we performed a sensitivity analysis to evaluate the effect of *D*_*tiss*_ on estimates of *D*_*wall*_(*t*).

## Results

### The effect of EP on permeability of blood vessels—experimental results

**Fig 4:**
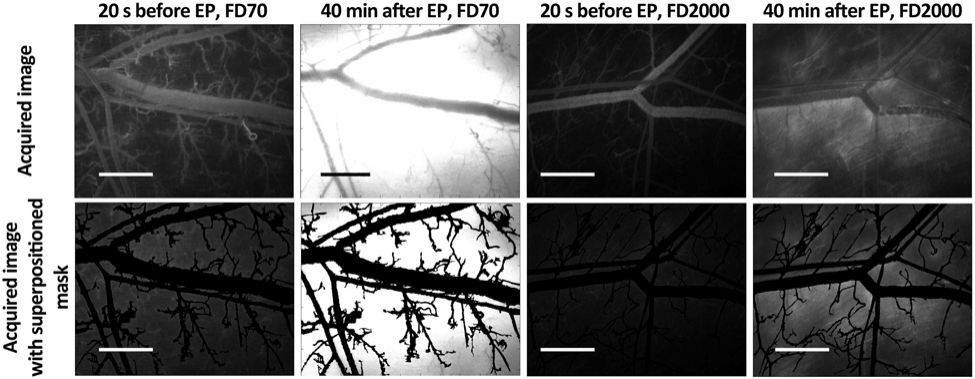
Fluorescent *in vivo* imaging of FD distribution in the tissue. Fluorescent *in vivo* imaging of FD distribution in the intra and extravascular space and superposition of the masks used for image analysis. Construction of masks of blood vessels networks enabled to discriminate between the FD that was present in small blood vessels and FD that extravasated into the tissue. Before electroporation (t = 20 s) there was no FD detected outside the blood vessels as seen when the masks were superpositioned on the original image (no FD fluorescence outside the tissue). When the masks were superpositioned on the original image at t = 40 min after EP a high increase of FD fluorescence intensity outside blood vessels was detected. FD70–70 kDa FD, FD2000–2000 kDa FD. The scale bar is 1 mm.

**Fig 5:**
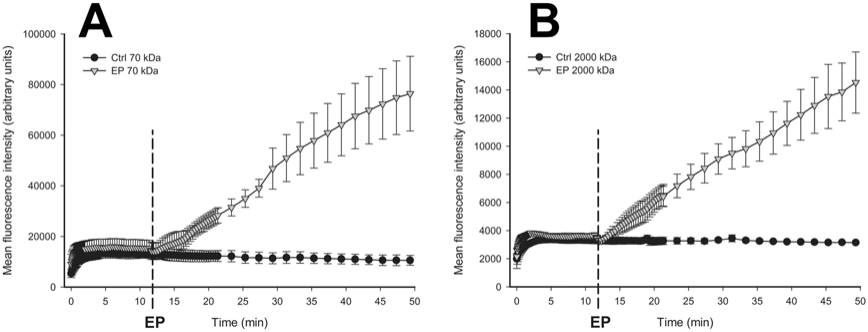
The temporal profile of the mean FD fluorescence intensity in the tissue. The temporal profile of the mean FD fluorescence intensity in the tissue (extravascular compartment) after application of EP for 70 kDa FD (A) and for 2000 kDa FD (B). Data represents mean values from 3 mice. Error bars represent SE. The span of x axis corresponds to Phases II and III form Fig. 1.

The comparison of fluorescence intensity of 70 kDa and 2000 kDa FD molecules visualized within the DWC is shown in Fig. 4. Images in upper panel illustrate the fluorescence of FD molecules acquired in the DWC before and after electroporation (i.e. 20 s before and 40 min after the delivery of EP pulses). The image acquired 20s before the delivery of EP pulses shows that the FD molecules within non-electroporated skin remain mainly confined to the intravascular compartment while the image acquired 40 min after the delivery of EP pulses shows that the FD molecules within the electroporated skin extravasate into the extravascular compartment. The illustration of the removal of the larger vessels (i.e. superimposed mask) from the images is given in the bottom panel. The areas remaining after removal of all larger vessels and their immediate surroundings represented the region of interest, comprising only the microcirculatory network and the extravascular space surrounding the microvessels. The masked images were used for determination of the mean fluorescence intensity in the treated tissue with respect to time (i.e. temporal profile of FD fluorescence intensity) as shown in Fig. 5.

After intraorbital injection of FD there was a rapid (within 5 s) increase in mean fluorescence intensity in the blood vessels and the tissue regardless of the size of the injected FD (Fig. 5A, B). With both sizes of FD, the mean fluorescence intensity in the tissue (extravascular compartment) was quickly increasing during the first ~2 min after the intraorbital injection and reached the maximum within 5 min. In all experimental groups the mean fluorescence intensity in the tissue started to decrease visibly within 10 min after the intraorbital injection of FD’s (Fig. 5). In the control groups this decrease continued during the entire remaining observation period. However, in the groups where EP pulses were applied, there was initially a small decrease in mean fluorescence intensity (which appeared to be more evident for 2000 kDa FD (Fig. 5B)), followed by a gradual and almost linear increase in mean fluorescence intensity over the remaining period of observation (FD 70 kDa—R^2^ = 0.8191; FD 2000 kDa—R^2^ = 0.8263) (Fig. 5A, B).

### The effect of EP on permeability of blood vessels—results of mathematical modeling

**Fig 6:**
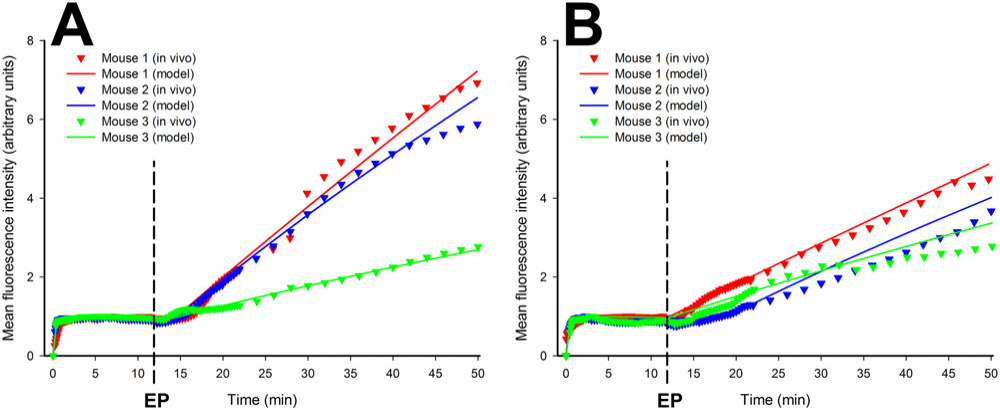
Mean fluorescence intensity vs. time curve fitting of *in vivo* experimental data. Mean fluorescence intensity vs. time curve fitting of *in vivo* experimental data for 70 kDa (A) and 2000 kDa (B) FD extravasation from the blood vessels. Triangles in A and in B represent data from *in vivo* experiments obtained for 70 kDa and 2000 kDa FD, respectively. The solid lines in both figures represent the time curve fitted data from the model for each mouse separately.

The *in vivo* experimental mean fluorescence intensity data and the results of modeling of the extravasation of FD from blood vessels are given in Fig. 6A and Fig. 6B for 70 kDa and 2000 kDa FD respectively. Each curve in Fig. 6 was normalized with respect to the maximum fluorescence intensity value reached before application of EP pulses within Phase II (see also Fig. 1).

The final values of the apparent diffusion coefficients of microvessel wall *D*_*wall*_ [μm^2^/s] for 70 kDa and 2000 kDa FD are given in Table 1. The *D*_*wall*_ values were calculated for the tissue diffusion coefficients *D*_*tiss*_ = 30 μm^2^/s and 5 μm^2^/s for 70 kDa and 2000 kDa FD molecular weights, respectively. In addition, the corresponding values of the apparent permeability coefficient *P*_*wall*_ (cm/s) were calculated according to the relation *P*_*wall*_ = *D*_*wall*_/*d*_*wall*_ [36] after replacing the unit of μm with cm and with *d*_*wall*_ representing the estimated microvascular wall thickness (Table 1).

**Table 1:** Calculated individual and median values of *D*_*wall*_ [μm^2^/s] and *P*_*wall*_ [cm/s10^-7^].

70 kDa FD	*D*_*wall*_	median *D*_*wall*_	*P*_*wall*_	median *P*_*wall*_
(*D*_*tiss*_ = 30 [μm^2^/s])	[μm/s]	[μm^2^/s]	[cm/s 10^-7^]	[cm/s10^-7^]
Mouse 1	0.0089		8.9	
Mouse 2	0.0086	**0.0086**	8.6	**8.6**
Mouse 3	0.0033		3.3	
**2000 kDa FD**	*D*_*wall*_	median *D*_*wall*_	*P*_*wall*_	median *P*_*wall*_
**(*D*_*tiss*_ = 5 [μm^2^/s])**	[μm^2^/s]	[μm^2^/s]	[cm/s 10^-7^]	[cm/s10^-7^]
Mouse 1	0.0047		4.7	
Mouse 2	0.0045	**0.0045**	4.5	**4.5**
Mouse 3	0.0038		3.8	

Calculated apparent diffusion coefficients *D*_*wall*_ [μm^2^/s], apparent permeability coefficients *P*_*wall*_ [cm/s10^-7^] and corresponding median values of *D*_*wall*_ and *P*_*wall*_ of electroporated microvessel wall for 70 kDa and 2000 kDa FD molecules

The calculated median values of apparent diffusion coefficients *D*_*wall*_ were 0.0086 μm^2^/s and 0.0045 μm^2^/s for 70 and 2000 kDa FD molecules, respectively. The apparent diffusion coefficient calculated for 70 kDa FD was almost twice (factor of 1.91) as large as the apparent diffusion coefficient for 2000 kDa FD. However, there was one outlier case for 70 kDa FD which for unknown reason did not fit well with the rest of the data (compare individual values of *D*_*wall*_ and *P*_*wall*_ in Table 1). This can also be observed in Fig. 6A, where the relative slope of increasing fluoresecence for mouse 3 for 70 kDa FD is clearly less steep, thus indicating poorer extravasation of FD, than those of other mice, probably due to a less-than-ideal application of EP pulses in this particular case.

**Fig 7:**
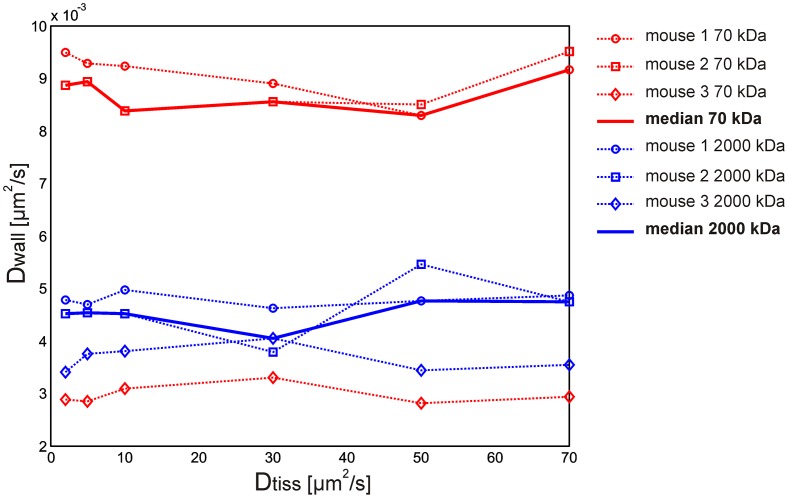
Sensitivity analysis of the calculated apparent diffusion coefficient *D*_*wall*_. Sensitivity analysis of apparent diffusion coefficient *D*_*wall*_ for 70 kDa and 2000 kDa FD to the values of *D*_*tiss*_ over the range from 2 μm^2^/s to 70 μm^2^/s. *D*_*wall*_(*D*_*tiss*_) functions calculated for 70 kDa and 2000 kDa FD are represented with red and blue curves, respectively. The bold curves represent the median values of *D*_*wall*_.

The results of sensitivity analysis showed that the calculated *D*_*wall*_ values were not sensitive to the values of the tissue diffusion coefficients *D*_*tiss*_ over a wide range from 2 μm^2^/s to 70 μm^2^/s (Fig. 7).

## Discussion and Conclusions

The objective of this work was to experimentally and mathematically investigate electroporation-facilitated *in vivo* extravasation of macromolecules from blood vessels into the surrounding tissue. *In vivo* extravasation of two different sizes of FDs (70 kDa and 2000 kDa) was investigated for an electroporation protocol used in clinical electrochemotherapy and clinical electrogene therapy of skin tumors: 8 square-wave electric pulses, voltage-to-distance ratio of 1300 V/cm, duration of 100 μs and frequency of 1 Hz [11][42]. FD molecules of two different sizes were used to simulate the extravasation of therapeutics of similar molecular weights [47–49] such as for example antibodies (size comparable to 70 kDa FD) and plasmid DNA (size comparable to 2000 kDa FD). However, it is important to note that 70 kDa FD is not a suitable model macromolecule to simulate the extravasation of albumin (66 kDa) in spite of similar molecular weights due to large difference in their hydrodynamic radii (i.e. 6.5 nm and 3.5 nm for 70 kDa FD and albumin, respectively) [50].

The extravasation was mathematically modeled as a transvascular diffusive transport of FD due to EP-induced increase in microvascular permeability. The transport across the electroporated microvessel wall was quantified by the determination of apparent diffusion coefficients *D*_*wall*_ [μm^2^/s] for both sizes of FDs. The qualifier "apparent" was used to reflect the unknown contribution of convection to the total extravasation of dextrans and hence to the estimated values of *D*_*wall*_. The apparent diffusion coefficient of non-electroporated microvessel wall was considered to be zero, since it has been previously shown that in normal conditions microvessels walls are hardly permeable to 70 kDa and 2000 kDa FD molecules [39,43,44].

The results of our study showed that the electroporation-induced permeability of microvessels was 1.91 times greater for smaller FDs (70 kDa) compared to larger ones (2000 kDa). The calculated median values of apparent diffusion coefficients were *D*_*wall*_ = 0.0086 μm^2^/s and *D*_*wall*_ = 0.0045 μm^2^/s for 70 and 2000 kDa FD, respectively (see Table 1 and Fig. 7). Our results are consistent with the previous findings on microvascular permeability for the macromolecules larger than 7 nm (molecular weight above 60 kDa) [51]. Namely, the permeability of continuous microvascular wall was found to rapidly decrease as molecular diameter increases from 0.5 to 5 nm and declines very slowly for molecules larger than 7 nm (i.e. at molecular weights above 60 kDa) [35,36,51,52]. These findings and the results of our study are indicating that the convection component is importantly involved in transvascular macromolecular transport after electroporation. Namely, the ratio of calculated apparent diffusion coefficients (i.e. 1.91) was lower than expected for free diffusion where the difference in hydrodynamic radius suggests a diffusion ratio of 4.14 based on the Stokes-Einstein equation (i.e. the inverse ratio of hydrodynamic radii of the two FD species being 26.89 nm for 2000 kDa and 6.49 nm for 70 kDa FD [50]).

As a result of our mathematical modeling and model parameter optimization we also determined that the transport across the electroporated microvascular wall needs to be characterized by a time dependent apparent diffusion coefficient *D*_*wall*_(t): a delayed linear functional dependency of *D*_*wall*_(t) (Fig. 3) resulted in best agreement between numerical calculations and experimental measurements *in vivo*. The time delay *T*_*del*_-*T*_*EP*_ between the delivery of EP pulses and the start of perceptible increase in apparent permeability of the vessel walls probably occurred due to the well-known effect of transient constriction of afferent blood vessels and to the corresponding blood flow modifications [19–21,23]. This is also in agreement with previous findings that the extravasation of larger FD molecules is delayed longer as opposed to the extravasation of the smaller FD molecules [23]. After the time delay *T*_*del*_-*T*_*EP*_, the increase in permeability of microvessel wall was not immediate but was modeled by a linearly increasing function (*T*_*del*_ < t < *T*_*sat*_) until the final saturation value of the apparent diffusion coefficient was reached *D*_*wall*_(t) = *D*_*wall*_ (t > *T*_*sat*_). We considered constant value of *D*_*wall*_ for t > *T*_*sat*_ since the *in vivo* image acquisition lasted for 40 min after electroporation and during this observation time, there was an almost linear increase of fluorescence intensity in the tissue (70 kDa FD—R^2^ = 0.8191; FD 2000 kDa—R^2^ = 0.8263R). Furthermore, current studies report that the time needed for the permeability of microvessel wall to return to the baseline is between 30 and 60 min after EP [21,23]. Our model was designed to describe the process occurring at early time points after electroporation, therefore it is not suitable for prediction of apparent diffusion coefficient at latter times (the gradual returning to its basal pre-electroporation value).

The apparent diffusion coefficient of the extravascular space *D*_*tiss*_ in our models was a constant value, which we estimated based on the data from the literature. We used the data published by Sykova and Nicholson [34] for brain tissue to estimate the values of *D*_*tiss*_ for 70 and 2000 kDa FD (Table 1), since the data of *D*_*tiss*_ for skin tissue [38][46] were available only for FD smaller than those examined in our study. We however performed sensitivity analysis to examine whether the calculated values of *D*_*wall*_ were sensitive to a selected value of *D*_*tiss*_ from the range of values found in literature (from 2 μm^2^/s to 70 μm^2^/s) and demonstrated that the calculated values of *D*_*wall*_ were not sensitive to the values of D_tiss_ (Fig. 7). The calculated *D*_*wall*_ was roughly tree orders of magnitude lower than the selected diffusion coefficient *D*_*tiss*,_ which is comparable to the ration between *D*_*wall*_ and *D*_*tiss*_ reported in the literature [32,53].

In addition, we compared the apparent diffusion coefficient of *D*_*wall*_ calculated in our study to the values of diffusion coefficients reported in the literature for microvessel walls with altered/increased permeability due to pathological changes such as inflammation [32] and cancer [37]. Kim and colleagues [32] assessed the possible changes in permeability of microvessel wall of hamster cheek pouch due to a topical application of calcium ionophore A23187 or bradykinin to elicit inflammation, which resulted in 2-fold increase of diffusion coefficients (for 70 KDa and 150 kDa dextrans) relative to their control values (i.e. normal tissue—no application of calcium ionophore A23187 and bradykinin to the tissue). The diffusion coefficients they determined for inflammation conditions were 0.00183 μm^2^/s (versus 0.0009 μm^2^/s for the control) and 0.00083 μm^2^/s (versus 0.00027 μm^2^/s for the control) for 70 kDa and 150 kDa dextrans, respectively. Our *D*_*wall*_ calculated for 70 kDa FD was therefore 4.7 times higher than the inflammation-increased diffusion coefficient determined by Kim and colleagues [32] (and an order of magnitude higher relative to their control value). The *D*_*wall*_ we calculated for 2000 kDa was 5.4 times higher than the value of the inflammation-increased diffusion coefficient determined for 150 kDa by Kim and colleagues [32] (and more than 16 time higher relative to their control although the 2000 kDa dextran molecule is 13 times larger molecule). Dreher and colleagues [37] measured tumor vascular permeability in solid tumors via a DWC model for FD’s with molecular weights from 3.3 kDa to 2000 kDa. The diffusion coefficient they determined for 2000 kDa was 0.0017 μm^2^/s, which is 2.65 times lower value than the *D*_*wall*_ of permeabilised microvessel wall by electroporation that we obtained in our study. The comparison of *D*_*wall*_ values determined in our study to the data reported by previous investigators indicates that the increase in blood vessel permeability due to electroporation can considerably enhance transvascular transport of macromolecules. Therefore, we can presume that distribution of molecules, whose diffusion into the tissues is limited, can be greatly enhanced by application of EP pulses, thus reaching more target cells.

To the best of our knowledge, this preliminary study is the first one to provide quantified values of apparent diffusion coefficient of electroporated microvessel wall for macromolecules with sizes of 70 kDa and 2000 kDa. These results may have important implications in therapies which require large therapeutic molecules to be transported across the blood vessel wall to the target cells. Our findings may also help understand the influence of both vascularization and vascular response to electroporation (i.e. increased local permeability of blood vessels due to EP pulses) on drug delivery and distribution within the target tissues, which is particularly important in electrochemotherapy, gene therapy and DNA vaccination where electroporation is used as a gene delivery method. The electroporation protocols used in different applications vary considerably; therefore further investigation of electroporation-facilitated extravasation is warranted for currently used protocols in order to optimize extravasation of therapeutics over a wide range of molecular sizes.

## Supporting Information

S1 Fig Schematic representation of two compartment model of FD pharmacokinetics. Schematic representation of two compartment model of FD uptake from orbital sinus (i.e. compartment 1) to blood and subsequent clearance of FD from blood (i.e. compartment 2); *m*_*orb*_ and *m*_*iv*_ denote the amount of FD in the compartments.
(TIF)

S1 Text The relationship between the average fluorescence *F(t)*, total amount of dextran *m(t)* and the average concentration of dextran *c(t)*. (DOC)

S2 Text Two-compartment pharmacokinetic model. (DOC)

S3 Text Finite element model of dextran transport. (DOC)
