# Supplementary material for: Modeling of Microvascular Permeability Changes after Electroporation
Source: PLoS One. 2015 Mar 20;10(3):e0121370. doi: 10.1371/journal.pone.0121370 (PMC4368817; doi:10.1371/journal.pone.0121370)
Supplement: S1 Text — (DOC) [file pone.0121370.s002.doc]

**The relationship between the average fluorescence *F(t)*, total amount of dextran *m(t)* and the average concentration of dextran *c(t)***

Intensity of fluorescence at each image location (pixel) was directly proportional to the amount (mass) of FD in the volume of tissue under this pixel. The total fluorescence intensity of all image locations (pixels) and also the average intensity of all pixels *F(t)* in the region of interest were therefore proportional to the total amount of FD within this region. Since the tissue was a single layer of skin of uniform thickness (estimated at 300 μm - see also Fig. 2), *F(t)* was also proportional to the average concentration of FD *c*(*t*) within the region of interest. The effect of light absorption by hemoglobin was considered negligible due to exclusion of all larger vessels from the images. The described linear relationships are given in Equations (S1) and (S2):

$m(t)=k_{m}\cdot F(t)$ (S1)

$c(t)=k_{c}\cdot F(t)$ (S2)

where *k_m_* and *k_c_* are unknown proportionality factors between *F*(*t*) expressed in arbitrary units (a.u.) and *m*(*t*) and *c*(*t*) expressed in (mol) and (mol/m^3^), respectively. The temporal profile of fluorescence intensity *F*(*t*) can therefore be used as a proxy for both *m*(*t*) or *c*(*t*) as input data for mathematical modeling of transport of FD from microvessels into the tissue and also within the tissue.
