# Supplementary material for: Modeling of Microvascular Permeability Changes after Electroporation
Source: PLoS One. 2015 Mar 20;10(3):e0121370. doi: 10.1371/journal.pone.0121370 (PMC4368817; doi:10.1371/journal.pone.0121370)
Supplement: S2 Text — (DOC) [file pone.0121370.s003.doc]

**Two-compartment pharmacokinetic model**

The dynamics of FD amount in the blood vessels was modeled using an open two-compartment pharmacokinetic model (S1 Fig) following three assumptions: 1. the FD molecules do not cross intact microvascular wall before the delivery of electric pulses; 2. the unknown volume of the compartments remains constant; and 3. the total volume of blood in the organism is sufficiently large compared to the volume of blood within the electroporated area so that the concentration of FD in fresh blood reaching the electroporated area remains unaffected by the amount of locally extravasated FD. The compartments 1 and 2 in S1 Fig represent the orbital sinus (site of injection) and intravascular space, respectively. The FD transfer between compartments 1 and 2 is unidirectional, since the orbital sinus acts as a reservoir of FD thus the concentration of FD in this compartment cannot be below the concentration in compartment 2. The *m_orb_*(*t*) in S1 Fig represents the amount of FD in the first compartment following an ideal bolus injection of the dose *m*_0_ injected at time *t = T_EP_*. The quantity *m_iv_*(*t*) in the second compartment represents the FD amount in blood vessels (i.e. intravascular compartment). The model is described by Equations (S3) and (S4) with *k*_12_ and *k*_20_ as constant transfer rate coefficients.

$\frac{dm_{orb}(t)}{dt}=-k_{12}\cdot m_{orb}(t); m_{orb}(T_{EP})=m_{0}; for t\geq T_{EP}$ (S3)

$\frac{dm_{iv}(t)}{dt}=k_{12}\cdot m_{orb}(t)-k_{20}\cdot m_{iv}(t); m_{iv}(T_{EP})=0; for t\geq T_{EP}$ (S4)

The solutions of Equations (S3) and (S4) are given in Equations (S5) and (S6), respectively.

$m_{orb}(t)= m_{0}\cdot e^{-k_{12}\cdot t}$ (S5)

$m_{iv}(t) = m_{0}\cdot\frac{k_{12}}{k_{12}-k_{20}}\cdot(e^{-k_{20}\cdot t}-e^{-k_{12}\cdot t})$ (S6)

The relationships between the amount and the concentration of FD in each of the compartments are:

$m_{iv}(t)= V_{iv}\cdot c_{iv}(t) \text{and} m_{orb}(t)= V_{orb}\cdot c_{orb}(t)$ (S7)

where the *V_orb_* and *V_iv_* stand for unknown but constant distribution volumes of FD for orbital sinus and intravascular compartment, respectively. By combining Equations (S6) and (S7) the temporal profile of FD concentration within the intravascular compartment (Compartment 2, S1 Fig) can be expressed as:

$c_{iv}(t) = c_{0}\cdot\frac{V_{orb}}{V_{iv}}\cdot\frac{k_{12}}{k_{12}-k_{20}}\cdot(e^{-k_{20}\cdot t}-e^{-k_{12}\cdot t})$, (S8)

The six unknown constants to the left of the exponential terms in Equation (S8) can be lumped into a single unknown constant coefficient *K* and Equation (S8) rewritten as:

$c_{iv}(t) = K\cdot(e^{-k_{20}\cdot t}-e^{-k_{12}\cdot t})$. (S9)

Values of parameters *k*_12_, *k*_20_ and *K* were determined by fitting Equation (S9) to experimental fluorescence data *F*(*t*) for Phase II (Fig. 1) separately for each individual experiment. The model optimization based on minimization of the sum of squares of the error was performed in Matlab software using its built-in optimization least-squares algorithm. The resulting temporal profiles of FD concentration in the intravascular compartment *c_iv_*(*t*) obtained from the optimized model for Phases II and III were used as input functions in the FEM of transvascular diffusive transport of FD.
