# Supplementary material for: Modeling of Microvascular Permeability Changes after Electroporation
Source: PLoS One. 2015 Mar 20;10(3):e0121370. doi: 10.1371/journal.pone.0121370 (PMC4368817; doi:10.1371/journal.pone.0121370)
Supplement: S3 Text — (DOC) [file pone.0121370.s004.doc]

**Finite element model of dextran transport**

The partial differential Equation describing Fick’s law is given in Equation (S10):

$\frac{\partial C(x,y,z,t)}{\partial t}-\nabla\cdot D(x,y,z,t)\cdot\nabla C(x,y,z,t)=0$. (S10)

where *C* [mol/m^3^] and *D* [m^2^/s] are the concentration and the apparent diffusion coefficient, of FD respectively and *x*, *y*, *z* and *t* stand for the spatial and temporal coordinates, respectively. Note that the spatially-dependent *C*(*x*,*y*,*z*,*t*) must not be confused with the spatially-independent average concentration *c*(*t*) from Equation (S2) or *c_iv_*(*t*) obtained from the pharmacokinetic model.

Within the model the structure of the skin was divided into two subdomains, the intravascular domain representing the capillaries, and the extravascular domain representing the surrounding interstitial tissue. The two subdomains were separated by a thin layer representing the capillary wall. The apparent diffusion coefficient of the capillary wall *D_wall_*(*t*) was modeled as a time-dependent function to reflect a delayed and gradual (not instantaneous) increase in transvascular permeability observed in experiments after application of EP pulses. On the other hand, the apparent diffusion coefficient of the extravascular space *D_tiss_* was fixed to a constant value. Different values of *D_tiss_* were adopted for 70 kDa and 2000 kDa FD based on the data reported for various tissues and sizes of FD [34,46]. A sensitivity analysis was performed to evaluate the sensititity of values of *D_wall_*(*t*) to the assumed value of *D_tiss_* over the range of values reported in the literature.

The solution of Equation (S10) over the appropriate geometry (described in the main text) provides spatial and temporal profile of FD concentration throughout the skin tissue model *C*(*x,y,z,t*). The total amount of FD *m*(*t*) (mol) within the tissue was obtained by integrating this spatially dependent concentration profile (mol/m^3^) over the volume of the modeled skin. The estimated concentration of the FD within the capillaries *c_iv_*(*t*) was incorporated into the FEM. The apparent diffusion coefficient of microvessel wall *D_wall_*(*t*) was obtained by fitting the FEM to the experimental data so that the temporal profile of FD content *m*(*t*) from the model best approximated the temporal profile of fluorescence intensity *F*(*t*).

The effect of the thin layer of capillary wall on trans-capillary diffusion of FD was modeled with the so-called ‘thin boundary layer condition’ option available in Comsol Multiphysics software. This option can be used to model a thin layer of a material with a dimension (thickness) much smaller than other parts of the model and is described with Equations (S11) and (12).

$-\mathbf{n}_{1}\cdot\left(D_{wall}(t)\nabla C(x,y,z,t)\right)_{1}=\frac{D_{wall}(t)}{w}\cdot\left(C_{1}(t)-C_{2}(t)\right)$ (S11)

$-\mathbf{n}_{2}\cdot\left(D_{wall}(t)\nabla C(x,y,z,t)\right)_{2}=\frac{D_{wall}(t)}{w}\cdot\left(C_{2}(t)-C_{1}(t)\right)$ (S12)

where **n**_1_ and **n_2_** are vectors normal to the inner and outer wall surface, respectively. Diffusion across the thin layer is possible in normal direction to the surface only, in our case normally to the capillary wall. *D_wall_*(*t*) is the apparent diffusion coefficient of the microvessel wall and *C*_1_(t) and *C*_2_(*t*) are FD concentrations at the inner and outer capillary wall surface, respectively. The FD concentration of the intravascular subdomain *c_iv_*(*t*) was calculated using Equation (S9). FEM method allows setting the concentration only at the boundaries and not inside the subdomains. To circumvent this limitation, we approximated a point-source of FD inside the capillaries by a small circle with radius *r*_1_ (rightmost zoomed circle in Fig. 2). The boundary condition assigned to this small circle was set to be equal to *c_iv_*(*t*). The diffusion coefficient within the capillary was assigned a very large value (10000 μm^2^/s) which was several orders of magnitude higher than what can be expected realistically, to achieve the effect of essentially instantaneous and homogeneous distribution of concentration of dextran inside the capillaries.

The insulation boundary conditions were applied at both outer surfaces of the skin layer geometry marked with number 1 (i.e. at the top and the bottom of the grayed-out element in indicating the surface of the skin and the glass layer of DWC), while the symmetry boundary conditions were applied at the inner boundaries marked with numbers 2 and 3 (Fig. 2). The insulation/symmetry boundary conditions are defined by Equation (S13):

$-\mathbf{n}\cdot\left(D_{tiss}\cdot\nabla C(x,y,z,t)\right)=0$ (S13)

where **n** is the normal vector to the boundary surface, *D_tiss_* is diffusion coefficient of the extravascular space, which was assumed to be constant and *C*(*x,y,z,t*) is the concentration of FD in the extravascular space.

The symmetry boundary conditions at the inner boundaries allowed us to simplify the geometry of the modeled skin layer. Namely, only the grayed-out region (i.e. the element n = N) of capillaries and the tissue (illustrated in Fig. 2) was actually modeled, while the white regions on the left ant right sides represent the infinite extension of the modeled geometry. This simplification reduced the computational time needed for numerical calculation and optimization process.
